# AQP3 Increases Intercellular Cohesion in NSCLC A549 Cell Spheroids through Exploratory Cell Protrusions

**DOI:** 10.3390/ijms22084287

**Published:** 2021-04-20

**Authors:** Sol Min, Chungyoul Choe, Sangho Roh

**Affiliations:** 1 Cellular Reprogramming and Embryo Biotechnology Laboratory, Dental Research Institute, School of Dentistry, Seoul National University, Seoul 08826, Korea; pine93@snu.ac.kr; 2 Samsung Medical Center, Samsung Biomedical Research Institute, School of Medicine, Sungkyunkwan University, Seoul 06351, Korea

**Keywords:** AQP3, aggregation, collective metastasis, NSCLC, lung cancer

## Abstract

Tumor cell aggregation is critical for cell survival following the loss of extracellular matrix attachment and dissemination. However, the underlying mechanotransduction of clustering solitary tumor cells is poorly understood, especially in non-small cell lung cancers (NSCLC). Here, we examined whether cell surface protrusions played an important role in facilitating the physical contact between floating cells detached from a substrate. We employed poly-2-hydroxyethyl methacrylate-based 3D culture methods to mimic in vivo tumor cell cluster formation. The suprastructural analysis of human NSCLC A549 cell spheroids showed that finger-like protrusions clung together via the actin cytoskeleton. Time-lapse holotomography demonstrated that the finger-like protrusions of free-floating cells in 3D culture displayed exploratory coalescence. Global gene expression analysis demonstrated that the genes in the organic hydroxyl transport were particularly enriched in the A549 cell spheroids. Particularly, the knockdown of the water channel aquaporin 3 gene (*AQP3*) impaired multicellular aggregate formation in 3D culture through the rearrangement of the actomyosin cytoskeleton. Moreover, the cells with reduced levels of AQP3 decreased their transmigration. Overall, these data indicate that cell detachment-upregulated *AQP3* contributes to cell surface protrusions through actomyosin cytoskeleton remodeling, causing the aggressive aggregation of free-floating cells dependent on the property of the substratum and collective metastasis.

## 1. Introduction

Lung cancer is the leading cause of cancer-related death worldwide; approximately 85% of all lung cancers are non-small cell lung cancers (NSCLC) [1,2,3,4]. Despite advances in early detection and standard treatment, NSCLC is often diagnosed at an advanced stage and with a poor prognosis; the overall cure and survival rate for NSCLC remains low at 19%, particularly in locally advanced stage IIIA cancer [5]. Even after complete primary tumor resection, about 45% of the early-stage NSCLC patients develop local recurrences or distant metastases within 8 to 18 months [6]. Therefore, the treatment and prevention of NSCLC can be improved with a better understanding of the biology and the mechanisms of metastasis.

Cancer metastasis is manifested by a highly complex cascade of processes, starting with the invasion of the tumor cells from a primary site into the surrounding tissues and continuing as intravasation into the circulatory system and extravasation to a distant organ, where the disseminated tumor cells that survive may initiate the progressive outgrowth of secondary tumors in a metastasis-receptive niche. However, metastasis is an inefficient process [7]. Approximately millions of cells per gram are disseminated from the primary tumors per day, but only a few become capable of transmigrating and surviving in a distant organ. One key limitation to successful metastasis is the death of the cells that occurs as they become detached from the extracellular matrix (ECM), which is known as anoikis, and from the neighboring cells, and undergo cell rounding, which is known as amorphosis [7,8].

Cancer cells have evolved multifaceted mechanisms, including the epithelial-to-mesenchymal transition (EMT), to safeguard against anoikis and amorphosis. Moreover, the recent work by several groups highlights the ability of the detached cells to form clusters or aggregates is another critical factor that can enhance the metastatic capacity of cancer cells [9,10,11]. Although metastasis has long been conceived of as a single-cell process, multicellular cell clusters, termed circulating tumor cell (CTC) clusters, of 2 to more than 10 cells tethered together have been directly observed in several steps of the metastasis cascade, including the systemic circulation of the tumor cells in the bloodstream. Aceto et al. showed that the CTC clusters appeared to be derived from the oligoclonal clumps of primary tumor cells rather than the coalescence of single CTCs in the circulation [9].

CTC clusters are associated with poorer prognoses in many cancer types [12]. Indeed, in different mouse models, multicellular aggregates give rise to between 50 and 97% of the metastases. The formation of clusters induces multiple molecular properties, including the increase in stem cell-like traits, evasion from targeting by natural killer cells, and resistance to metabolic stress, among others. However, the underlying spatiotemporal mechanism by which the detached cells tether together to form aggregates is poorly understood. According to several studies, canonical cell adhesion proteins, including cadherin, are involved in cancer cell cluster formation [13,14]. In addition, plakoglobin was shown to hold CTCs together [9]. However, these studies were mostly performed under adhesive 2D and 3D conditions that could not replicate the in vivo tumor microenvironment that became stiffer during the progression toward advanced cancer.

Here, we examined spatiotemporal cell interaction in the in vivo cancer pathological context by employing nonadhesive 3D poly-2-hydroxyethyl methacrylate (poly-HEMA) culture. The 3D cultures of cancer cells in poly-HEMA hydrogel, which prevents cell spreading and cell attachment to the substratum due to its superhydrophilic nature, have been used for many years to mimic in vitro 3D cancer tissue architecture, as cell aggregates in poly-HEMA are pathologically similar to clusters isolated from a patient’s CTC, ascitic fluid and pleural effusion [15,16,17]. We report that the clustering of free-floating NSCLC A549 cells in nonadhesive 3D poly-HEMA culture depends on actin-rich protrusions, which is intensely studied for cell migration under 2D culture conditions [18]. Furthermore, our current study is the first to indicate that AQP3, a unique member of the water channel aquaporin (AQP) family [19], is essential for forming protrusions by acting as a key regulator of actomyosin cytoskeleton remodeling through caspase 3 activation. We also discuss the implications of these findings in the context of multicellular metastasis in a hydrodynamic tumor microenvironment.

## 2. Results and Discussion

### 2.1. Detachment of NSCLC A549 Cells Leads to Protrusion Formation

**Figure 1:**
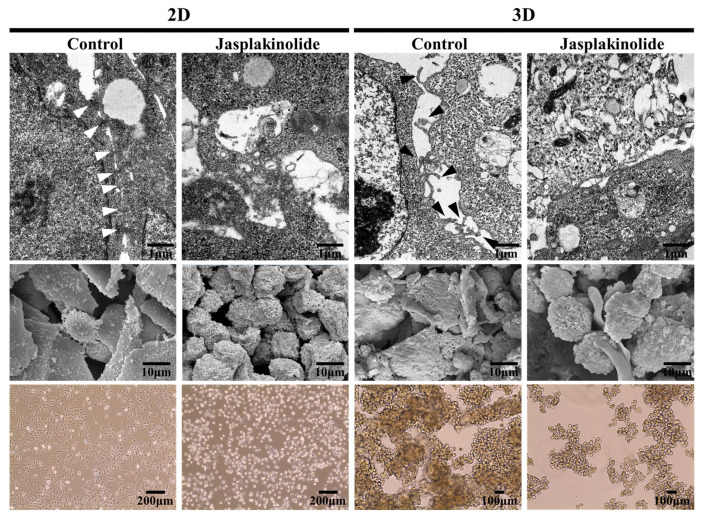
Ultrastructural analysis of non-small cell lung cancer (NSCLC) A549 cell spheroids. Representative images of NSCLC A549 cells showing cell surface architecture in a 2D adhesive culture dish (**left panel**) and a 3D poly-HEMA-coated dish (**right panel**). The images were taken after 3 days of culturing. The cells in the 2D monolayer culture were treated with 1 µM jasplakinolide for 1 h. Then, the cells were cultured on 2D tissue culture dishes or poly-HEMA-coated dishes. Upper panel: The representative transmission electron microscopy images of NSCLC A549 cells grown in 2D (**left**) and 3D (**right**) cultures. Middle panel: The representative scanning electron microscopy images of NSCLC A549 cells grown in 2D (**left**) and 3D (**right**) cultures. Lower panel: The phase-contrast micrographs showing the morphologies of human NSCLC A549 cells grown in 2D (**left**) and 3D (**right**) cultures. White arrowhead indicates tight junction; Black arrowhead indicates protrusion. Note the adherens junctions and protrusions. Original magnification: ×1000. Scale bar: 200 μm.

Our initial interest was to understand the dissociation of strongly refractory tumor spheroids into single cells by trypsin/ethylenediaminetetraacetic acid. This observation led us to hypothesize that structures different from canonical cellular adherens junctions exist to form tight spheroids. We identified the underlying structure organizing the spheroids by analyzing the cell surfaces at the nanometer resolution with a transmission electron microscope (TEM). TEM images showed that monolayer cells grown under 2D adhesive culture displayed tight connections with canonical adherens junction-like structures (Figure 1, upper panel). In contrast, A549 cell spheroids exhibited protrusions from the overall cell surface but no adherens junction-like structures. Interestingly, scanning electron microscope (SEM) analysis revealed that jasplakinolide, an actin stabilizer, increased plasma membrane tension in 2D monolayer cells, as evidenced by cell rounding (Figure 1, middle panel). Importantly, jasplakinolide impaired the formation of A549 cell spheroids, which are devoid of protrusions, suggesting that cell surface extensions were actomyosin-dependent (Figure 1, bottom panel). Together, these findings reveal that protrusions are critical cell surface suprastructures connecting cells in 3D tumor spheroids in a manner depending on the actin cytoskeletons. 

### 2.2. Clustering of Solitary Human NSCLC A549 Cells Is an Active Process

**Figure 2:**
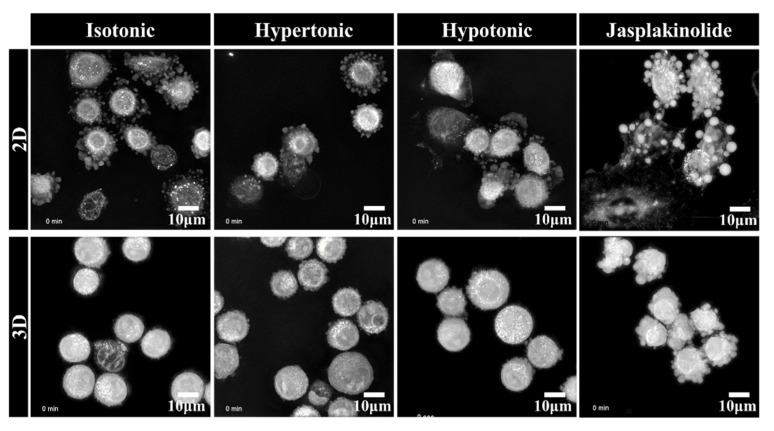
Representative live-cell three-dimensional holotomography of NSCLC A549 cells. The cells were exposed to 30% deionized water and 5% sucrose to give an osmotic shock to induce hypotonic stress and hypertonic stress, respectively. Images were taken for 10 h upon seeding the cells. The cells in the 3D poly-HEMA-coated nonadhesive dishes (lower panel) show exploratory protrusions compared to the cells in the 2D normal adhesive culture dish (upper panel). Additionally, see Supplementary Movies 1 and 2.

The self-organizing capacity of solid tumor cells to construct 3D cellular structures was long considered a passive process: deposited on substrata where the spreading is energetically unfavorable, cells autonomously form clusters that compact due to surface tension [20]. We further examined how the protrusions interacted with each other to form the spheroids using time-lapse holotomographic microscopy, which provided information on the spatiotemporal organization of cells in 3D. The cells were analyzed upon seeding on an adhesive 2D substrate or a nonadhesive poly-HEMA 3D hydrogel. Interestingly, the detached cells occurring early upon seeding in adhesive 2D culture exhibited extension (Figure 2, Supplementary Movie 1) that was not detected in the cells attached to the substratum in a TEM image (Figure 1). Unlike the protrusions in the cell aggregates, larger protrusions in the suspended cells on a 2D adhesive substratum are likely bleb-like structures since jasplakinolide does not change the protrusions. Larger blebs have been observed in amoeboid and lobopoid cell migration [21].

In contrast, A549 cell spheroids showed the radial sprouting of finger-like structures that were enriched with actin (Figure 2, Supplementary Movie 1). Notably, the protrusions were very dynamic, with cells moving around and repeatedly coalescing and repulsing, suggesting their importance for self-organization. It is also worth noting that the blebs disappeared after the cells attached to the substratum, whereas the finger-like protrusions in the spheroids were preserved after clustering. Furthermore, the cells exposed to osmotic stress demonstrated changes in protrusions and cell contraction, suggesting that protrusions responded dynamically to physiological changes in the hydrodynamic microenvironment. These results indicate that the finger-like protrusions in A549 cell spheroids actively cling together and form differential spheroids in response to the diverse tumor microenvironment in relation to the osmotic and hydrostatic interstitial fluid pressures and ECM stiffness.

### 2.3. Upregulation of Hydrostatic Pressure-Regulated Genes in NSCLC A549 Spheroids

Several lines of evidence suggest that protrusions are dynamically controlled by the interplay of hydrostatic pressure and actomyosin cytoskeleton remodeling [22,23]. Actin-free membrane blebs are primarily controlled by hydrostatic pressure [18,24]. In contrast, finger-like protrusions accompany cofilin-mediated depolymerization and *de novo* actomyosin assembly, which requires the actin nucleators Arp2/3 complex and formin mDia1, and Rho GTPase signaling [25,26,27].

**Figure 3:**
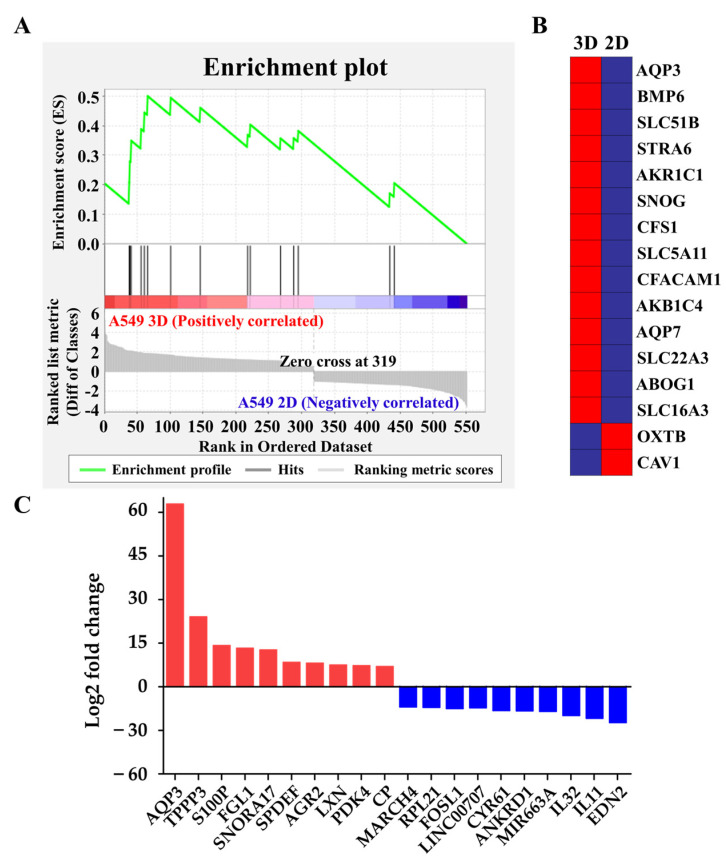
Global gene expression profiling. (**A**) The enrichment plot of the gene set of organic hydroxyl transport by GSEA. Bottom panel: the plot of the ranked list of all genes, with the Y-axis indicating the value of the ranking metric and the X-axis indicating the rank for all the genes. The genes whose expression levels are most closely associated with the A549 spheroid group have the highest metric scores with positive or negative signs and are located at the left or right edge of the list. Middle panel: the location of the genes from organic hydroxyl transport within the ranked list. Top panel: the running enrichment score for the gene set as the analysis walks along with the ranked list. The score at the peak of the plot is the enrichment score (ES) for this gene set. The genes that appear before or at the peak are defined as core enrichment genes for this gene set. (**B**) The heat map of the core enrichment genes corresponding to A. The genes that contribute most to the ES, i.e., genes that appear in the ranked list before or at the peak point of ES, are defined as core enrichment genes. Rows, genes; columns, samples. The range of colors, from red to blue, indicates the range of expression values, from high to low, respectively. (**C**) The expression levels (log2 fold change (fc); A549 spheroid fc/A549 2D fc) of top-10 upregulated and downregulated genes.

**Figure 4:**
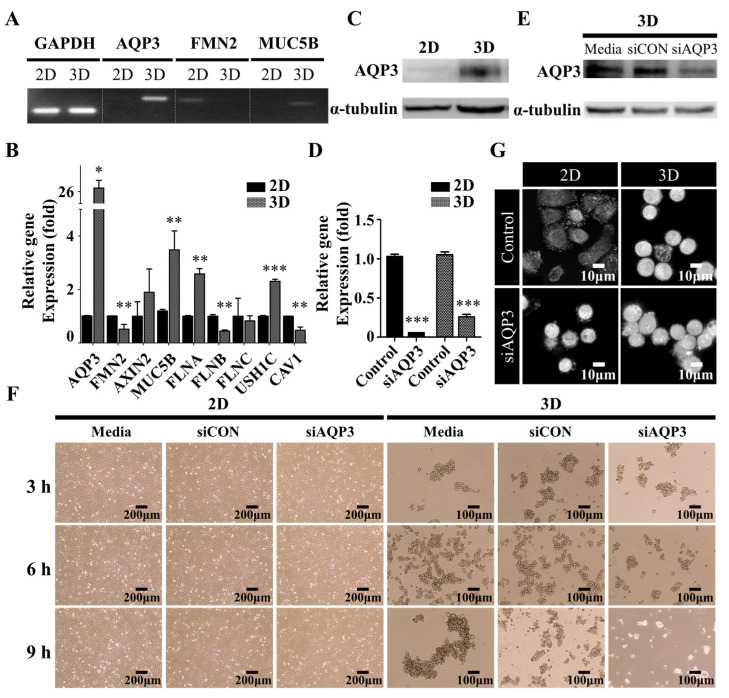
The effect of AQP3 on spatiotemporal dynamics of protrusions. (**A**) Polymerase chain reaction and (**B**) Quantitative real-time reverse transcription-polymerase chain reaction of the transcript levels of the organic hydroxyl transport genes. The data shown here represent three independent experiments, and the values represent the mean ± SEM of triplicate samples. The level of each mRNA was normalized to that of the GAPDH mRNA in the same sample and presented as the fold-change over that of the 2D culture control cells. The differences in expression levels were evaluated for significance using two-tailed t-tests with unequal variance. * *p* < 0.05; ** *p* < 0.01; and *** *p* < 0.001. (**C**) The Western blot of AQP3 levels in 2D and 3D culture cells. The A549 cells were harvested following twenty-four h seeding to confirm AQP3 protein levels. α-tubulin was used as an internal control. (**D**) The downregulation of *AQP3* following transfection of *AQP3* siRNA. The A549 cells were pre-transfected for twenty-four h, and further incubated for twenty-four h to confirm the knockdown of *AQP3* mRNA levels. The level of each mRNA was normalized to that of the GAPDH mRNA in the same sample and presented as the fold-change over that of the each of control groups. (**E**) The Western blot of AQP3 in 3D culture cells following transfection of *AQP3* siRNA. Twenty-four h following siRNA transfection, the A549 cells were harvested to confirm the knockdown of *AQP3* by evaluating the AQP3 protein levels with Western blotting. α-tubulin was used as an internal control. (**F**) The phase-contrast micrograph showing the morphologies of the A549 cells grown in 2D (left) and 3D (right) cultures. The micrograph (**F**) and representative live-cell three-dimensional holotomography (**G**) of the A549 cells showing the effect of *AQP3* knockdown on the growth behavior of the A549 cells in 2D (left) and 3D (right) cultures. Twenty-four h following siRNA transfection in the 2D culture, the cells were further incubated in the 2D or 3D culture condition. Additionally, see Supplementary Movie 1 and Supplementary Movie.

We further explored the underlying molecular mechanism by which A549 cells switched from extruding blebs to the actin-rich finger-like protrusions depending on their substratum by performing RNA-sequencing profiling and gene set enrichment analysis (GSEA) to evaluate the global transcriptomic changes associated with protrusions. Interestingly, we found that the gene sets of organic hydroxyl transport were significantly enriched in A549 spheroids (Figure 3A,B); this observation supported our hypothesis that hydrodynamic mechanisms were critical for protrusion formation in the A549 cell spheroids [22,23]. We also investigated the expression levels (log2 fold change) of top-10 upregulated and downregulated between A549 spheroids and 2D A549 cells, revealing that *AQP3* is one of the most markedly increased genes in A549 spheroids (Figure 3C, Table 1). Polymerase chain reaction (PCR) and quantitative real-time reverse transcription-polymerase chain reaction (qRT-PCR) quantification further confirmed the expression levels of several potential signature genes for protrusion, including formin 2 (*FMN2*) [28] and mucin 5b (*MUC5B*) [29] (Figure 4A,B). A549 spheroids exhibited elevated *AQP3* mRNA and AQP3 protein levels (Figure 4A–C). From an evaluation of each candidate gene in the GSEA and the fold change comparison, *AQP3* was of special interest. More importantly, the cancer genome atlas (TCGA) showed that *AQP3* in NSCLC patients undergo higher genomic alteration than other types of cancer (Supplementary Table S1). In light of the fact that genomic alteration is the underlying cause of tumor development, we conclude that AQP3 plays a significant role in the context of tumor pathology.

AQP3, in contrast to most members of the water channel aquaporin (AQP) family, can transport other small molecules, such as glycerol and H2O2, important for the physiological and pathological balance of hydrostatic and osmotic pressures in the plasma membrane [30]. To examine its role in the protrusion formation, LUAD A549 cells were transfected with *AQP3* siRNA to knock down its expression. The expression of *AQP3* was found to be markedly downregulated by this siRNA, as verified by qRT-PCR quantification and Western blotting (Figure 4D,E). Consistent with the expectation, we found that the knockdown of *AQP3* attenuated protrusion formation, resulting in less compact aggregates (Figure 4F,G). These data together suggested that the water channel protein AQP3 critically influenced the spatiotemporal dynamics of protrusions.

### 2.4. Downregulation of AQP3 Gene Expression Results in Cortical Actomyosin Remodeling

Based on the result showing that A549 cells treated with actin stabilizer jasplakinolide attenuated protrusion formation (Figure 1), we hypothesized that AQP3 could control cluster formation through actomyosin in suspended culture condition. Cell rounding rearranges the cytoskeleton architecture from cell body stress fiber to cortical actomyosin, which is regulated by Rho-associated protein kinases (ROCK). In addition, cell rounding forms cortical actin in response to hydrostatic pressure induced by actomyosin contraction, resulting in the inhibition of cell shrinkage and cell death [22,23,31].

**Figure 5:**
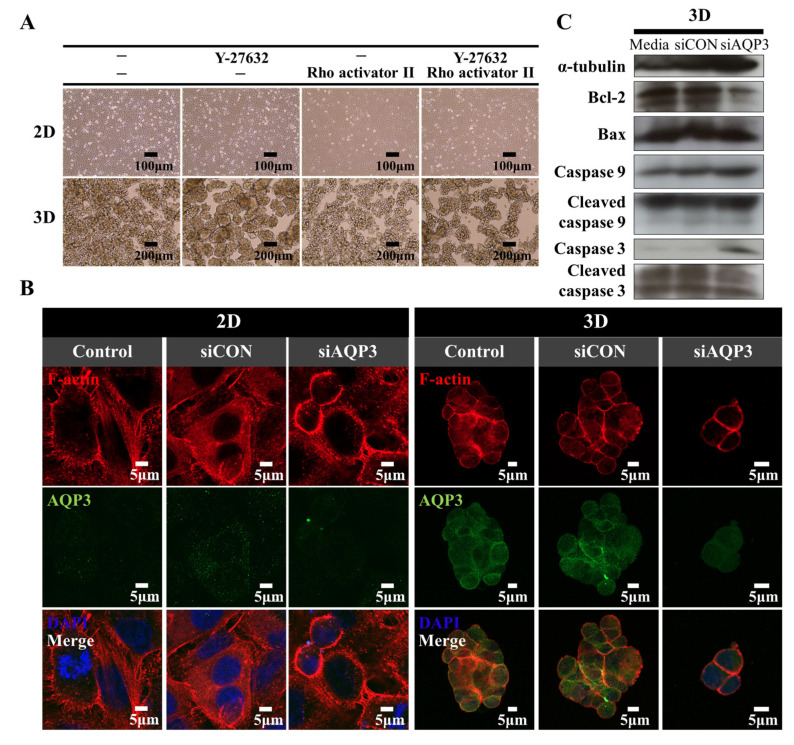
A549 cell aggregation is an active process. (**A**) The effect of ROCK on A549 cell aggregation. The phase-contrast micrographs show the morphologies of the A549 cells following treatment with a ROCK inhibitor, Y-27632, and the Rho activator Ⅱ. (**B**) The effects of *AQP3* knockdown with siRNA on actomyosin cytoskeleton remodeling. A549 cells were stained with anti-AQP3 antibody, followed by Fluorescein-conjugated antibody (green). The actin microfilaments were stained with rhodamine-conjugated phalloidin (red), and the nuclei were stained with DAPI (blue). (**C**) The effects of *AQP3* knockdown with siRNA on the apoptosis signaling pathway. Twenty-four h following siRNA transfection in 2D culture condition, the cells were further incubated in the 2D or 3D culture condition. Then, cells were harvested to confirm the effect of *AQP3* knockdown on the apoptotic molecular signatures using Western blotting. α-tubulin was used as an internal control.

Thus, we first examined whether the protrusions in the cells under attached and suspended culture conditions were regulated by ROCK. The cells were treated with ROCK inhibitor Y-27632 and Rho activator CNF toxins. Y-27632 increased the growth of A549 cell spheroids, whereas CNF toxins attenuated the growth of A549 cell spheroids (Figure 5A). In contrast, A549 cells in 2D culture did not exhibit changes in cell growth patterns. These results suggest that ROCK actively regulates A549 cell spheroids through the protrusions. We then investigated whether AQP3 regulated actomyosin rearrangement. The cells transfected with *AQP3* siRNA exhibited cell shrinkage and peripheral actomyosin compared to the control siRNA-treated cells. These data suggest that AQP3 plays a key role in protrusion formation under 3D culture conditions (Figure 5B). ROCK activity is regulated by apoptosis. Interestingly, A549 spheroids with downregulated *AQP3* unexpectedly exhibited an increased level of anti-apoptotic marker Bcl-2 along with the increase of proapoptotic caspase 3 and 9, suggesting AQP3 regulated actomyosin cytoskeleton remodeling through the caspase pathway (Figure 5C). We further investigated the actomyosin remodeling mechanism of AQP3 in other NSCLC cell line H460. While knockdown of *AQP3* with *AQP3* siRNA in H460 cultured in adhesive monolayer did not influence actomyosin remodeling, downregulation of AQP3 under the 3D culture condition significantly decreased the aggregation formation and rearranged actomyosin (Supplementary Figure S1). This result suggests that the actomyosin remodeling could be the underlying mechanism by which AQP3 controls the fate of NSCLC tumor cells following substratum detachment. While the current study did not demonstrate the mechanism by which AQP3 affected myosin II activation downstream of caspase 3, it nevertheless confirmed that *AQP3* expression in cell spheroids contributed to the generation of protrusions through the apoptotic signaling pathway.

### 2.5. Protrusion Controls Invasion in A549 Cancer Cells 

**Figure 6:**
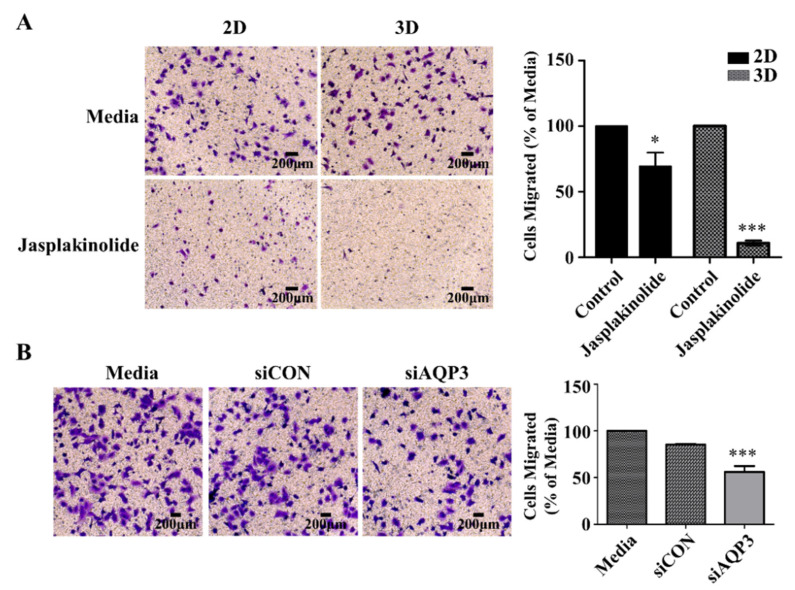
Protrusion controls migration and invasion of A549 cancer cells. (**A**) The phase-contrast micrograph of the migration of NSCLC A549 cells (left) and quantification (right) in the Transwell assay. The quantification of migrated cells treated with jasplakinolide represents three independent experiments, and the values represent the mean ± SEM of triplicate samples. The differences in expression levels were evaluated for significance using unpaired two-tailed t-test. * *p* < 0.05; ** *p* < 0.01; and *** *p* < 0.001. (**B**) Representative images (left) and quantification (right) of A549 spheroid transfected with siRNA *AQP3*. Twenty-four h following siRNA transfection in 2D culture condition, the cells were further incubated in the 2D or 3D culture condition. The migration capacity of the A549 spheroids with a knocked down level of AQP3 was much lower than that of the negative control cells. The differences in expression levels were evaluated for significance using one-way ANOVA followed by Tukey’s post-hoc tests. * *p* < 0.05; ** *p* < 0.01; and *** *p* < 0.001.

In addition to protrusions’ role in tying cells in A549 cell spheroids, we asked whether they played an important role in cell migration. Single-cell migration has been extensively studied on adhesive 2D surfaces. However, it remains unclear how aggregated cells manage to migrate for invasion and metastasis, especially in a 3D cancer environment [32,33,34,35]. Therefore, we investigated whether the protrusions of A549 spheroids could control spheroid migration. We employed a Boyden chamber to compare the migration of the cells incubated in media, the A549 cells on a 2D adhesive surface, and the A549 spheroids treated with the actin stabilizer jasplakinolide. (Figure 6A). Interestingly, A549 spheroids with reduced levels of AQP3 showed a decrease in migration (Figure 6B). In light of previous results that jasplakinolide and AQP3 influence protrusion, these results suggest that protrusion plays an important role in collective integrin-independent migration.

## 3. Materials and Methods

### 3.1. Cell Culture and Reagents

The human pulmonary adenocarcinoma A549 cells (The Korean Cell Line Bank, Seoul, Korea) of a human alveolar basal epithelial carcinoma cell line were maintained in Roswell Park Memorial Institute (RPMI-1640) medium supplemented with 10% fetal bovine serum (Gibco, Grand Island, NY, USA). The cells were cultured at 37 °C under a humidified atmosphere with 95% air and 5% CO_2_. Jasplakinolide and Y-27632 (Cayman Chemical, Ann Arbor, MI, USA) dissolved in dimethyl sulfoxide (Sigma-Aldrich, St. Louis, MO, USA) to reach a concentration of 1 mM. Rho activator II was obtained from Cytoskeleton Inc. (Denver, CO, USA). The cells were exposed to 30% deionized water and 5% sucrose to give an osmotic shock to induce hypotonic stress and hypertonic stress, respectively.

### 3.2. Imaging

Holotomographic images of the cells were taken on the 3D Cell-Explorer Fluo (Nanolive, Ecublens, Switzerland) using a low-power class I laser (0.2 mW/mm^2^, λ = 520 nm), a 60 × dry objective (NA = 0.8), and a USB 3.0 CMOS Sony IMX174 sensor with a typical quantum efficiency of 70% at 545 nm, dark noise (typical) of 6.6 e^−^, and a typical dynamic range of 73.7 dB. In the holotomographic image, the lateral (X and Y-axis) resolution was 200 nm, the Z-axis resolution was 400 nm, with a field of view of 90 × 90 × 30 µm, and the maximum temporal resolution was 0.5 fps 3D RI volume per second. 

### 3.3. Time-Lapse Imaging

Live cell imaging was conducted in a Top-Stage Incubator system (Okolab, Pozzuoli, Italy) at 37 °C with 5% CO_2_ and humidifying conditions. The cells were cultured in FluoroDish cell culture dishes (World Precision Instruments Inc., Sarasota, FL, USA) for this experiment.

### 3.4. Image Analysis

Image rendering and export were performed with the STEVE v.1.7.3496 software (Nanolive). The backgrounds were subtracted during post-processing, and all the slices of the post-processed image were exported to RI volumes and transformed into the 3D tiff format. The RI volumes in the tiff format can be read by the software FIJI. Three-dimensional RI volumes of all the slices were transformed into 2D RI maps using maximum intensity projection and exported to a time-lapse video file.

### 3.5. Poly-HEMA Coating

First, 1.2 g of poly-HEMA (Sigma-Aldrich) was dissolved in 40 mL of 95% ethanol by mixing the solution overnight at 37 °C. Then, 50 μL or 3.2 mL of the poly-HEMA stock solution were added to 96-well plates and 10-cm dishes, respectively, under the tissue culture hood; the plates and dishes were swirled for 10 min using a plate rotator. The plates were left to dry overnight and then washed with phosphate-buffered saline (PBS) immediately before use. 

### 3.6. RNA Sequencing

Total RNAs were isolated from different cell lines using Trizol (Invitrogen, Carlsbad, CA, USA). Total RNA quantity and quality were verified spectrophotometrically (Nano-Drop 2000 spectrometer; Thermo Scientific, Wilmington, DC, USA) and electrophoretically (Bioanalyzer 2100; Agilent Technologies, Palo Alto, CA, USA). To prepare Illumina-compatible libraries, a TruSeq RNA library preparation kit (Illumina, San Diego, CA, USA) was used according to the manufacturer’s instructions. In brief, mRNA purified from total RNA using polyA selection was chemically fragmented (50-bp fragment libraries) and converted into single-stranded cDNA using random hexamer priming. After this, the second strand was generated to create double-stranded cDNA that was ready for TruSeq library construction. Short double-stranded cDNA fragments were then connected with sequencing adapters, and suitable fragments were separated by agarose gel electrophoresis. Truseq RNA libraries were built by PCR amplification and quantified using quantitative PCR (qPCR) according to the qPCR Quantification Protocol Guide. qPCR data were qualified using the Agilent Technologies 2100 Bioanalyzer (Agilent technologies). Libraries were sequenced (101-nt paired-end sequencing) using a HiSeq™ 2000 platform (Illumina). To estimate expression levels, the RNA-Seq reads were mapped to the human genome using TopHat (version 1.3.3) [36]. The reference genome sequence (hg19, Genome Reference Consortium GRCh37) and annotation data were downloaded from the UCSC website (http://genome.uscs.edu (accessed on 15 April 2021)). The transcript counts at the gene level were calculated, and the relative transcript abundances were measured in fragments per kilobase of transcript per million mapped reads (FPKM) using Cufflinks software (version 1.2.1; Seattle, WA, USA) [37]. FPKM is computed similarly to RPKM, except it accounts for the scenario in which only one end of a pair-end read is mapped [38]. Using this approach, the expression levels were measured for 37,396 Ref-Seq genes uniquely aligned based on RNA sequencing reads. Raw data were extracted as FPKM values across all samples, and samples with zero values across more than 50% of uniquely aligned genes were excluded.

### 3.7. siRNA-Mediated Knockdown of AQP3

The transient knockdown of *AQP3* was performed using Lipofectamine^TM^ RNAiMAX (ThermoFisher, Rockford, IL, USA). The cells were plated in a 6-well plate at a density of 3 × 10^5^ cells per well and cultured overnight at 37 °C. The following day, the cells were transfected with *AQP3* siRNA (sequence available in Supplementary Table S2) or non-targeting control siRNA (OriGene, Rockville, MD, USA) using 7.5 μL of Lipofectamine^TM^ RNAiMAX according to the manufacturer’s instructions. The final concentration of the siRNA used per well was 25 pmol. After incubating for 24 h, the cells were divided into conventional 2D and poly-HEMA 3D cultures and incubated further for 24 h for the following experiments. 

### 3.8. qRT-PCR

Total RNA was extracted from the cultured cells using the PureLink^TM^ RNA Mini Kit (Invitrogen). The first-strand cDNA was synthesized using oligo-dT primers and M-MLV reverse transcriptase (Invitrogen). qRT-PCR reactions were performed in triplicates at a final volume of 20 μL containing TB Green Premix Ex Taq II (Takara, Shiga, Japan), 10 ng of cDNA, and 20 pmol of each primer. qRT-PCR was performed using a 7500 real-time PCR system (Applied Biosystems, Foster City, CA, USA) at 95 °C for 30 s, followed by 40 cycles of 95 °C for 5 s and 60 °C for 34 s. The glyceraldehyde 3-phosphate dehydrogenase gene (*GAPDH*) was used as an internal control in each reaction. Specific amplification was verified by performing a melting curve analysis (55–95 °C, 0.5 °C/s). The quantification of relative gene expressions was performed using the ΔΔCT method. The expression level of each gene was normalized to that of *GAPDH* in the same sample. Genes and their primers are listed in Supplementary Table S3. 

### 3.9. Western Blot Analysis

Cells were lysed with RIPA buffer (Santa Cruz Biotechnology, Santa Cruz, CA, USA) on ice for 30 min, and the lysates were centrifuged at 13,000 g at 4 °C for 15 min. The supernatants were incubated with 4 × Laemmli sample buffer (Bio-Rad, Hercules, CA, USA) at 95 °C for 5 min. The samples were then separated with SDS-PAGE gel and immunoblotted with the antibody against AQP3 (Alomone Labs Ltd., Jerusalem, Israel, 1/200), GAPDH (BioLegend, San Diego, CA, USA), or β-actin (Santa Cruz Biotechnology) or α-tubulin (Santa Cruz Biotechnology). β-actin, GAPDH, and α-tubulin were used as loading controls.

### 3.10. Immunocytochemistry

A549 cells were seeded on sterile glass coverslips, and immunocytochemical staining was performed. In short, the cells on coverslips were fixed with 4% paraformaldehyde for 10 min and permeabilized with 0.15% Triton-X 100 for 5 min. Then, the cells were blocked for 1 h with the blocking solution of 3% bovine serum albumin in PBS and incubated with the primary antibody against AQP3 for 2 h at room temperature. Subsequently, the cells were incubated with Fluorescein-conjugated anti-rabbit IgG (Sigma-Aldrich) for 60 min at room temperature. The subcellular organization of the actin microfilaments was assessed by incubating the cells with rhodamine-conjugated phalloidin (Molecular Probes, Eugene, OR, USA) at a dilution of 1:200 to reach the final concentration of 1.5 units/mL. Next, the cells were washed with PBS, and the coverslips were mounted on a glass slide in 10% Mowiol 4–88, 1 μg/mL 4′,6-diamidine-2-phenylindole dihydrochloride, and 25% glycerol in PBS with nuclei counterstained blue with 4′,6-diamidine-2-phenylindole dihydrochloride (DAPI). Then, the cells were observed under a confocal laser scanning microscope LSM800 (Zeiss, Oberkochen, Germany). 

### 3.11. Scanning Electron Microscopy of Spheroids 

The cell spheroids were collected using wide pipette tips and pooled into an Eppendorf tube. Following a PBS wash, the spheroids were incubated overnight in 2.5% glutaraldehyde (EMS, Hatfield, PA, USA), 1.25% paraformaldehyde (EMS), and 0.03% picric acid in 0.1 M sodium cacodylate buffer (pH 7.4) at 4 °C. The spheroids were then washed in 0.1 M cacodylate and post-fixed with 1% osmium tetroxide (OsO4)/1.5% potassium ferrocyanide (KFeCN6) for 1 h. The samples were then washed 2 times in PBS, dehydrated with ethanol, exposed to critical-point drying, placed on glass coverslips, and subjected to platinum sputtering before imaging. Images were acquired at 20 kV at 1000–1500× magnification using scanning electron microscopy (JSM 630/OA, JEOL Ltd., Tokyo, Japan).

### 3.12. Transmission Electron Microscopy of Spheroids 

The fixed spheroids that were serially dehydrated with ethanol were subsequently infiltrated by a mixture of ethanol and propylene oxide at the ratio of 2:1, 1:1, 1:2, or 0:1 for 1 h, and then by a mixture of propylene oxide and epoxy resin (Structure Probe, Inc., West Chester, PA, USA) at the ratio of 2:1, 1:1, or 1:2 for 1 h. Then, the spheroids were embedded in epoxy resin and loaded into capsules to be polymerized at 60 °C for 72 h. Following the staining of the semi-fine thin 1-µm sections with toluidine and sodium tetraborate, thin-sectioning at 80 nm was performed using a Leica EM UC7 ultramicrotome (Leica Microsystems, Wetzlar, Germany). The resulting sections were collected on copper grids and contrasted in 1% uranyl acetate solution in distilled water for 1 h at room temperature in the dark and lead citrate. The images were acquired using a JEM-1400 Flash TEM (JEOL Ltd.) at 120 kV. 

### 3.13. Boyden Chamber Assay 

The migration of A549 cells was examined using a 6.5 mm Transwell (Corning, Glendale, AZ, USA). The cells were plated on the inserts and cultured at 37 °C in the upper chambers. After 20 h, the migrated cells that had crossed the inserts were fixed with 4% paraformaldehyde for 15 min and stained with 0.1% crystal violet (Sigma-Aldrich) for 10 min. The inserts were washed at least three times in PBS and the interior of the inserts was gently swabbed with a cotton swab to remove the non migrated cells. Then, the migrated cells counted as cells per field of view under phase-contrast microscopy.

## 4. Conclusions

Our study demonstrates that cell detachment-induced *AQP3* upregulation contributes to the extrusion of the cell surface to form protrusions through caspase 3, leading to the differential aggregation of substratum-detached cells important for multicellular metastasis in a manner dependent on the properties of the substratum.

The significance of our study is two-fold. First, there is increasing evidence showing that multicellular tumor cell aggregates are critical for cell survival following the loss of ECM attachment and dissemination through the circulatory system. The current study demonstrates that AQP3 contributes to tumor cell clustering through cell surface membrane protrusion. The AQP family comprises 13 mammalian members. While they primarily facilitate the passive transport of water across membranes, they also play a crucial role in tissue migration during embryonic development and wound healing. Furthermore, several studies have reported that this unexpected role for AQPs in cell migration is also implicated in tumor cell migration [30,39,40,41,42]. Chae and colleagues reported that AQP5 promoted tumor invasion in NSCLC. However, the mechanism underlying the AQP5-mediated invasion has not been delineated. Indeed, our study is the first to elucidate the mechanism of AQP3 in influencing multicellular aggregation through protrusion-promoted coalescence under suspended cell growth conditions.

Second, protrusions have been extensively studied in tissue regeneration, cancer invasion and metastasis, and the environmental exploration of leukocytes [43,44,45]. However, many in vitro studies are performed with cells in adhesive flat 2D culture, under which integrin-mediated adhesion to the ECM is preserved. However, cancer invasion and metastasis occur independently of cell adhesion to ECM, as evidenced by pathological clusters isolated from patients’ CTCs, ascitic fluid, and pleural effusion [15]. A study using intravital imaging reported that CTCs with active transforming growth factor-β (TGF-β) signaling migrate as solitary cells, whereas the cells lacking TGF-β signaling invade lymphatics collectively, suggesting that TGF-β signaling regulates the mode of cancer cell motility [46]. However, the mechanisms underlying tumor aggregate formation under cancer pathological conditions remain poorly studied. Indeed, our results demonstrate for the first time that protrusions are important in 2D cellular movement and also play a critical part in the 3D aggregates of cancer cells detached from the substratum via the downstream apoptosis executor caspase 3 and migration.

Finally, it will be interesting to elucidate the mechanism through which protrusions contribute to cell-cell cohesion in cancer clusters following substratum detachment. Considering the studies demonstrating that hydrostatic or osmotic pressure controls cell rounding, we hypothesize that protrusion-mediated cell aggregation under suspension conditions proceeds in two steps, that protrusions should first render floating cells migratory and then adhesive. We propose that hydrostatic pressure built up locally through AQP3 channels that extrude through cell surface protrusions, acting as pedals in a fluid environment and increasing intercellular interactions to overcome Brownian dispersion. Consistent with this proposal, Saadoum et al. reported that as the underlying mechanism by which non-endothelial cells overexpressing *AQP1* or *AQP4* showed accelerated cell migration, the AQPs at the protrusions at the leading edge led to rapid water fluxes, providing the space for actomyosin assembly and flow [42]. However, it is still unclear how protrusions are adhesive and contribute to inducing the self-assembly of the floating cells. Cell clustering in directed multicellular migration can be subdivided into cohort aggregates, in which the cells are in tight contact with each other, or streaming aggregates, in which the coordinated aggregation is not always in direct physical contact [34]. Although our study did not definitely confirm the type of aggregates in the A549 cell spheroids, it would be interesting to investigate the molecular components and viscoelastic properties of the protrusions. Addressing this idea in detail is beyond the scope of the current study. Furthermore, our findings strongly support the idea that protrusions are a useful target in anticancer drug development, particularly targeting advanced lung cancer characterized by highly motile EMT. However, the details of the spatiotemporal architecture of the protrusions in the A549 cells and the localization of AQP3 in protrusions remain to be examined.

## Figures and Tables

**Table 1:** Fold changes in top-10 upregulated and downregulated genes.

Gene Symbol	Gene Description	^1^ Fold Change(log2)
*AQP3*	Aquaporin 3 (Gill blood group)	62.82
*TPPP3*	Tubulin polymerization-promoting protein family member 3	24.03
*S100P*	S100 calcium binding protein P	14.16
*FGL1*	Fibrinogen-like 1	13.25
*SNORA17*	Small nucleolar RNA, H/ACA box 17	12.62
*SPDEF*	SAM pointed domain containing ETS transcription factor	8.35
*AGR2*	Anterior gradient 2	8.11
*LXN*	Latexin	7.43
*PDK4*	Pyruvate dehydrogenase kinase, isozyme 4	7.20
*CP*	Ceruloplasmin (ferroxidase)	6.94
*MARCH4*	Membrane-associated ring finger (C3HC4) 4, E3 ubiquitin protein ligase	−6.93
*RPL21*	Ribosomal protein L21	−7.08
*FOSL1*	Fos-related antigen 1 isoform 2	−7.22
*LINC00707*	Long intergenic non-protein coding RNA 707	−7.49
*CYR61*	Cysteine-rich, angiogenic inducer, 61	−8.17
*ANKRD1*	Ankyrin repeat domain 1 (cardiac muscle)	−8.26
*MIR663A*	MicroRNA 663a	−8.46
*IL32*	Interleukin 32	−9.88
*IL11*	Interleukin 11	−10.85
*EDN2*	Endothelin 2	−12.28

^1^ Fold changes were calculated as the ratio of A549 spheroids to A549 2D cells.

## Data Availability

Not applicable.

## Supplementary Materials

The following are available online at https://www.mdpi.com/article/10.3390/ijms22084287/s1, Table S1: Copy number variation (CNV) of the 28 different tumor types, Table S2: Sequence information on the siRNAs used in this study, Table S3: Primers used in this study, Figure S1: The effects of *AQP3* siRNA knockdown on actomyosin remodeling in NSCLC cell line H460, Video S1: Live-cell holotomography of NSCLC A549 cells cultured in 2D or 3D culture, Video S2: Live-cell holotomography of NSCLC A549 cells following *AQP3 siRNA* transfection or treated with jasplakinolide.

## Abbreviations

AQP	Aquaporin
AQP3	Aquaporin 3
CAV1	Caveolin 1
CTC	Circulating tumor cell
ECM	Extracellular matrix
EMT	Epithelial-to-mesenchymal transition
ES	Enrichment score
FLNA	Filamin A
FLNB	Filamin B
FLNC	Filamin C
FMN2	Formin 2
FPKM	Fragments per kilobase of transcript per million mapped reads
GAPDH	Glyceraldehyde 3-phosphate dehydrogenase
GSEA	Gene set enrichment analysis
MUC5B	Mucin 5B
NSCLC	Non-small cell lung cancers
PBS	Phosphate buffered saline
PCR	Polymerase chain reaction
poly-HEMA	poly-2-hydroxyethyl methacrylate
qRT-PCR	Quantitative real-time reverse transcription-polymerase chain reaction
ROCK	Rho-associated protein kinases
RPMI	Roswell Park Memorial Institute
SEM	Scanning electron microscope
TEM	Transmission electron microscope
TGF-β	Transforming growth factor-β
USH1C	Usher Syndrome 1C
