# Supplementary material for: AQP3 Increases Intercellular Cohesion in NSCLC A549 Cell Spheroids through Exploratory Cell Protrusions
Source: Int J Mol Sci. 2021 Apr 20;22(8):4287. doi: 10.3390/ijms22084287 (PMC8074759; doi:10.3390/ijms22084287)
Supplement: Supplementary file 1 [file ijms-22-04287-s001.zip › Supplementary Tables.pdf]

Supplementary Table S1. Copy number variation (CNV) of the 28 different tumor types*

TCGA ID**	Tumor type	Case numbers		
		Total	Gain cases/ %	Loss cases/ %
LUAC	Lung squamous cell carcinoma	502	89/17.73	12/2.33
OV	Ovarian serous cystadenocarcinoma	585	75/12.82	22/3.76
ESCA	Esophagealcarcinoma	184	18/9.78	5/2.72
HNSC	Head and neck squamous cell carcinoma	521	42/8.06	14/2.69
STAD	Stomach adenocarcinoma	452	39/9.03	6/1.39
SARC	Sarcoma	260	16/6.15	11/4.23
LUAD	Lung adenocarcinoma	513	46/8.97	5/0.97
BLCA	Bladder urothelial carcinoma	408	29/7.11	11/2.7
MESO	Mesothelioma	84	2/2.38	6/7.14
SKCM	Skin cutaneous melanoma	468	25/5.34	16/3.42
CHOL	Cholangiocarcinoma	36	3/8.33	
GBM	Glioblastoma multiforme	596	16/2.68	28/4.70
UCS	Uterine carcinosarcomas	56	2/3.57	2/3.57
BRCA	Breast invasive carcinoma	1072	48/4.48	19/1.77
ACC	Adrenocortical carcinoma	90	3/3.33	2/2.22
PAAD	Pancreatic adenocarcinoma	175	8/4.57	1/0.57
UCEC	Uterine corpus endometrial carcinoma	510	19/3.73	5/0.98
LIHC	Liver hepatocellular carcinoma	371	13/3.50	3/0.81
DLBC	Diffuse large B-cell lymphoma	47	1/2.31	
PRAD	Prostate adenocarcinoma	487	3/0.62	6/1.23

*Data were downloaded from the TCGA portal
(https://portal.gdc.cancer.gov/genes/ENSG00000165272?canDistTable_offset=10)
** listed in order of highest to lowest CNV

Supplementary Table S2. Sequence information on the siRNAs used in this study

**Name**	**Sequence**
siAQP3 (1)	GGA GUG AAG UCA GGU CAU AAG UU TC
siAQP3 (2)	GGA GCA GUG GGA CGU GUU UCU GU CA
siAQP3 (3)	GCA AGG GAC CAG UCG GAA GGG AU TC
siNegative	UUC UCC GAA CGU GUC ACG UTT<br>ACG UGA CAC GUU CGG AGA ATT
	UGA CCU CAA CUA CAU GGU UTT<br>AAC CAU GUA GUU GAG GUC ATT

Supplementary Table S3. Primers used in this study

Gene	Name	Forward sequence (5' to 3')	Reverse sequence (5' to 3')
AQP3	Aquaporin 3	CCGTGACCTTTGCCATGTGC	TTGTCGGCGAAGTGCCAGAT
FMN2	Formin 2	GTTTCCTAGGCGAGTTCCATCC	CTTCTGGACAGCATCTGAGCGT
AXIN2	Axin 2	CAAACTTTCGCCAACCGTGGTTG	GGTGCAAAGACATAGCCAGAACC
MUC5B	Mucin 5B	CTGCTACGACAAGGACGGAAAC	AAGGCTGTGAGCGCACTGGATG
FLNA	Filamin A	CAACAAGTTCACTGTGGAGACCA	TGTAGGTGCCAGCCTCATAAGG
FLNB	Filamin B	CCTTCAAGGTGGCTGTCACTGA	CCCTCAACAGTTATGCCAAGCC
FLNC	Filamin C	ATGGTAGCTGCACCGTGGAGTA	TCCACCACATCCTTCACTGGCA
USH1C	Usher Syndrome 1C	TGTCTGCTGAGGTGGGATTGGA	CTGCGGCTACTCTTCAGCACAT
SLC5A11	Solute Carrier Family 5 Member 11	CCAAACTCGTGCTGGAACTCCT	GTGAAGATGGTGCTGGCACTGT
SLC51B	Solute Carrier Family 51 Subunit beta	ATGGTCCTCCTGGGAAGAAGCA	GCCTCATCCAAATGCAGGACTTC
CAV1	Caveolin 1	CCAAGGAGATCGACCTGGTCA	GCCGTCAAAACTGTGTGTCCCT
GAPDH	Glyceraldehyde-3-phosphate dehydrogenase	GTTCCAATATGATTCCACCC	GAAGATGGTGATGGGATTT
