# Supplementary material for: AQP3 Increases Intercellular Cohesion in NSCLC A549 Cell Spheroids through Exploratory Cell Protrusions
Source: Int J Mol Sci. 2021 Apr 20;22(8):4287. doi: 10.3390/ijms22084287 (PMC8074759; doi:10.3390/ijms22084287)
Supplement: Supplementary file 1 [file ijms-22-04287-s001.zip › Supplementary Figure 1.pdf]

Figure S1. The effects of AQP3 siRNA knockdown on actomyosin remodeling in NSCLC cell line H460

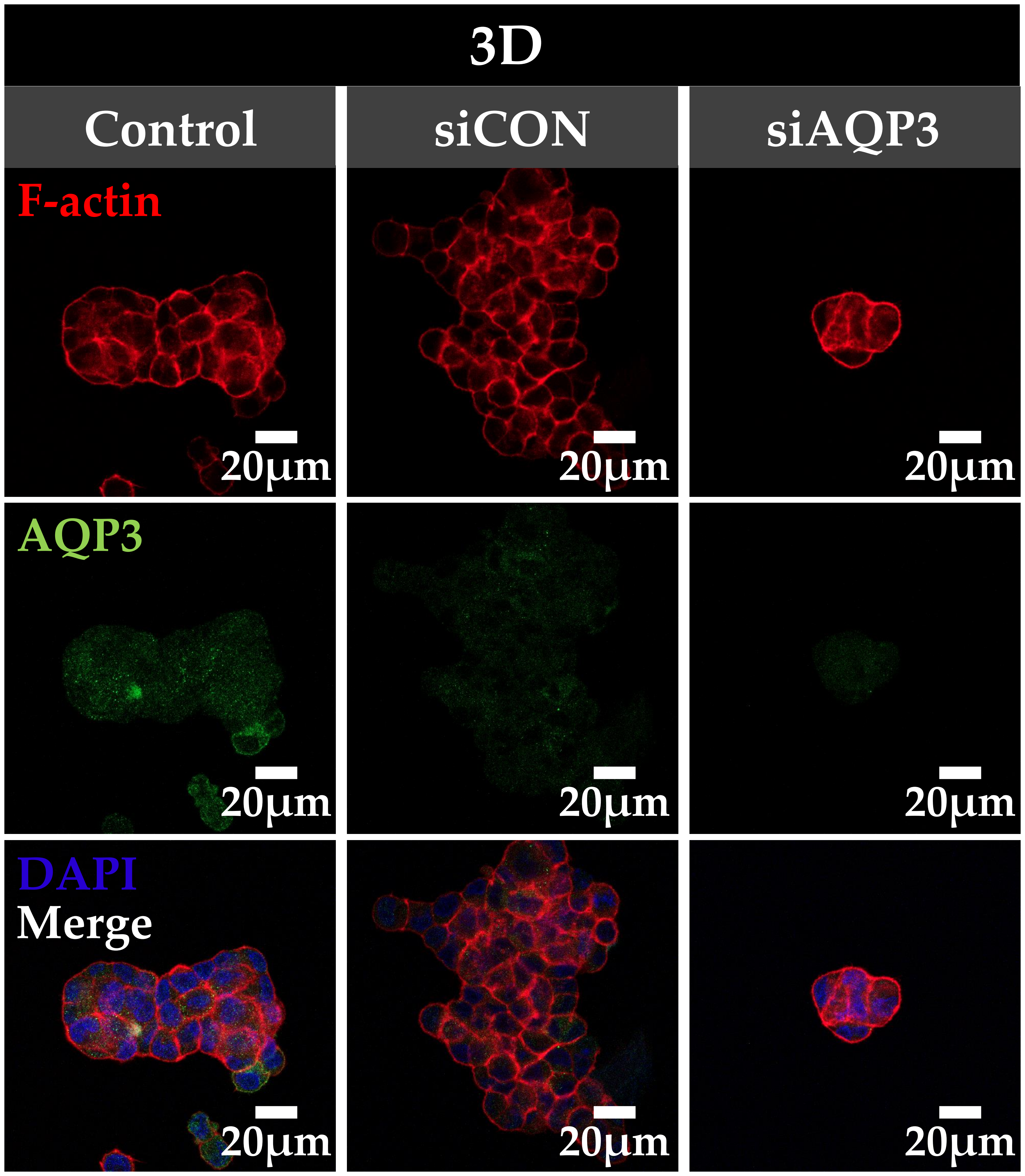

Figure S1. The effects of AQP3 knockdown with siRNA on actomyosin cytoskeleton remodeling. NSCLC H460 cells were stained with anti-AQP3 antibody, followed by Fluorescein-conjugated antibody (green). The actin microfilaments were stained with rhodamine-conjugated phalloidin (red), and the nuclei were stained with DAPI (blue).
